# Rituximab in Refractory Myasthenia Gravis: Experience in a Single Healthcare Center in Mexico

**DOI:** 10.7759/cureus.13226

**Published:** 2021-02-08

**Authors:** Juan Carlos López-Hernández, Javier A Galnares-Olalde, Enrique Gómez-Figueroa, Adib Jorge de Sarachaga, Edwin Steven Vargas-Cañas

**Affiliations:** 1 Neuromuscular Diseases, Instituto Nacional de Neurología y Neurocirugía Manuel Velasco Suárez, Mexico City, MEX; 2 Neurology, Instituto Nacional de Neurología y Neurocirugía Manuel Velasco Suárez, Mexico City, MEX

**Keywords:** myasthenia gravis (mg), refractory, prednisone, rituximab

## Abstract

Background: Ten to fifteen percent of patients with myasthenia gravis (MG) have treatment-refractory disease. In short series and case reports, rituximab has proven to be effective in refractory MG.

Methods: A retrospective, longitudinal study was conducted. Recruitment was performed in an MG cohort from a single third-level healthcare center in Mexico. The selection included refractory MG patients that were treated with rituximab. Response after rituximab therapy was assessed with MG composite score (MGCS) and prednisone dose reduction at 6, 12, and 18 months after initiation. Wilcoxon signed-rank test was used to evaluate differences between related groups for non-continual variables. P<0.05 was considered statistically significant.

Results: Ten patients (7%) fulfilled criteria for refractory MG, and eight of them were treated with rituximab. The mean age at MG diagnosis was 25.5 (±2) years, with a female predominance (75%). All our patients (100%) had positive acetylcholine receptor (AchR) antibodies. The median MG duration was six years (interquartile range [IQR] 4.2-6) before rituximab initiation. All patients were previously treated with azathioprine and 50% additionally with cyclophosphamide. The median prednisone doses before rituximab treatment and 18-month follow-up were 50 mg (IQR 30-50 mg) and 10 mg (IQR 0-20 mg), respectively (p=0.011).* *The median baseline MGCS and at 18-month follow-up were 19.5 (IQR 11-31) and 6 (IQR0-16), respectively (p = 0.012).

Conclusion: Rituximab appears to be associated with clinical improvement and prednisone dose reduction in Latin-American patients diagnosed with anti-AchR MG. Our findings need to be interpreted in light of the limitations mentioned.

## Introduction

Myasthenia gravis (MG) is an antibody-mediated autoimmune disease characterized by fatigable muscle weakness. It is the most frequent neuromuscular junction disorder, with an overall prevalence rate of 15-179 per million individuals [1]. Eighty percent of MG patients have antibody production against the nicotinic acetylcholine receptor (AchR) in the neuromuscular junction, and a smaller number of patients have antibodies against muscle-specific kinase (MusK) or lipoprotein-receptor-related protein 4 (LRP4). MG is classified into purely ocular or generalized, being the latter the most common subtype [2].

Recommended therapy for generalized MG includes steroids and immunosuppressant agents, such as azathioprine, cyclosporine, cyclophosphamide, and mycophenolate. Plasma exchange (PE) and intravenous immunoglobulin (IVIG) are recommended for myasthenic crisis, as well as severe presentations with bulbar muscle involvement, before thymectomy and to improve MG weakness before starting pharmacological therapies that take longer before an effect is observed. Both PE and IVIG are highly effective therapies but are also expensive [3,4].

Ten to fifty percent of MG patients are treatment refractory. Treatment-refractory MG is defined as a failure to respond adequately to conventional therapies, including insufficient response to maximal safe steroid dose and at least one immunosuppressive drug at an adequate dose and titration. Also, refractory MG is considered when relapses occur while reducing immunosuppressive therapy [2-5].

Rituximab is a humanized monoclonal antibody that binds to CD20 antigen, inducing complement (or antibody) mediated cytolysis. It has been proposed to be an effective therapy for refractory MG [6]. There is limited data on the clinical benefit and durability of rituximab treatment in refractory MG in Latin America. This study presents the experience with rituximab therapy in patients with refractory MG in a third-level healthcare center in Mexico.

## Materials and methods

An observational, longitudinal retrospective study was conducted. Recruitment was performed in an MG cohort from a single third-level healthcare center in Mexico. The selection included refractory MG that were treated with rituximab. Minimum follow-up duration after rituximab therapy initiation was 18 months. After selection, basal and subsequent clinical characteristics were collected to analyze clinical response to rituximab therapy.

The following data were collected: age at diagnosis, gender, Myasthenia Gravis Foundation of America Clinical Classification (MGFACC) at diagnosis, MG antibody serostatus, MG duration, comorbidities, thymectomy, myasthenic crisis before rituximab therapy, previous crisis treatment, previous immunosuppressive treatment, immunosuppressive dose, duration, and prednisone dose before treatment.

To assess outcome after rituximab therapy, MG composite score (MGCS) and prednisone dose reduction were evaluated at rituximab initiation and follow-up consult at 6, 12, and 18 months after initial therapy. Moreover, the presence/absence of myasthenic crisis and Myasthenia Gravis Foundation of America (MGFA) postintervention clinical status were also evaluated at 18 months' follow-up consult [7,8].

The rituximab dosing schedule used was an initial 1-gram intravenous infusion repeated two weeks after, followed by a 1-gram maintenance dose every six months depending on clinical response. All patients signed an informed consent, and the present study was approved by the ethics committee.

Statistical analysis: Categorical variables were described with frequencies, whereas continuous variables were described with mean, median, interquartile range, and min-max. Wilcoxon signed-rank test was used to evaluate differences between related groups for non-continuous variables. P<0.05 was considered statistically significant.

## Results

A total of 141 patients were diagnosed with MG in our institution. Ten patients (7%) fulfilled criteria for refractory MG, and eight of them were treated with rituximab. The baseline characteristics of our patients are listed in Table 1.

**Table 1:** Patient's characteristics MGFACC: Myasthenia Gravis Foundation of America Clinical Classification, MG: myasthenia gravis; AchR: Acetylcholine receptor; SLE: systemic lupus erythematosus; IVIG: intravenous immunoglobulin; PE: plasma exchange. AQP4: aquaporin 4; AZA: azathioprine; CP: Cyclophosphamide; RTX: Rituximab; MGCS; Myasthenia gravis composite score; N/A: not administered; MM-3: minimal manifestations.

	1	2	3	4	5	6	7	8
Age at diagnosis of MG (years)	35	53	24	19	17	15	25	16
Sex	M	M	F	F	F	F	F	F
MGFACC at diagnosis of MG	IIB	IIB	IIB	V	V	V	IIB	IIB
Serostatus	AchR	AchR	AchR	AchR	AchR	AchR	AchR	AchR
MG duration, (years)	8	3	6	4	9	8	6	5
Comorbidities	None	None	None	None	SLE Sjogren Anti-AQP4 (+)	None	None	None
Thymectomy	No	No	No	No	No	No	No	No
Myasthenic crisis before RTX (n)	3	2	1	1	4	4	3	4
Treatment for myasthenic crisis	IVIG	IVIG, PE	IVIG	PE	IVIG, PE	IVIG, PE	IVIG	IVIG, PE
Previous immunosuppressive treatment	AZA	AZA	AZA, CP	AZA	AZA, CP	AZA, CP	AZA, CP	AZA, CP
Immunosuppressive duration (months)	48	15	AZA: 12 CP: 12	12	AZA: 24 CP: 12	AZA: 24 CP: 12	36	AZA: 12 CP: 12
Prednisone dose pre-RTX	70 mg	80 mg	30 mg	35 mg	50 mg	40 mg	50 mg	50 mg
Prednisone dose 6-month post-RTX	40 mg	10 mg	10 mg	20 mg	40 mg	30 mg	25 mg	20 mg
Prednisone dose 12-month post-RTX	10 mg	None	5 mg	5 mg	20 mg	20 mg	40 mg	10 mg
Prednisone dose 18-month post-RTX	10 mg	None	None	None	10 mg	10 mg	20 mg	10 mg
MGCS pre-RTX	19	20	11	13	21	20	31	18
MGCS 6-month post-RTX	9	0	0	6	14	5	21	8
MGCS 12-month post-RTX	3	0	0	4	8	5	27	6
MGCS 18-month post-RTX	3	0	0	0	4	4	16	4
Myasthenic crisis since RTX	0	0	0	0	1	0	0	0
Time without relapses (months)	18	18	24	18	20	24	12	4
MGFA postintervention status	Improved to MM3	Improved to MM3	Improved to MM3	Improved to MM3	Improved to MM3	Improved to MM3	Improved to MM3	Improved to MM3

The mean age at MG diagnosis was 25.5 (± 2) years, with a female predominance (75%). Sixty-three percent of patients had MGFA class IIb and the rest of the patients had class V. One hundred percent of our patients had positive AchR antibodies. The median MG duration was six years (interquartile range [IQR] 4.2-6) before rituximab initiation. Only one patient had significant comorbidities, being diagnosed also with systemic lupus erythematosus (SLE), Sjogren syndrome, and positive antiaquaporin-4 antibodies (AQP4). None of the patients had thymoma in CT scans. All patients had a history of myasthenic crisis, with a median of 3 (IQR 1-4). Before rituximab treatment, all patients were previously treated with azathioprine and 50% additionally with cyclophosphamide. Quality of life in our patients was reported as poor. None of our patients had a thymectomy performed. The reason for this was that none of our patients had optimal conditions and MG control prior to surgical evaluation.

**Figure 1:**
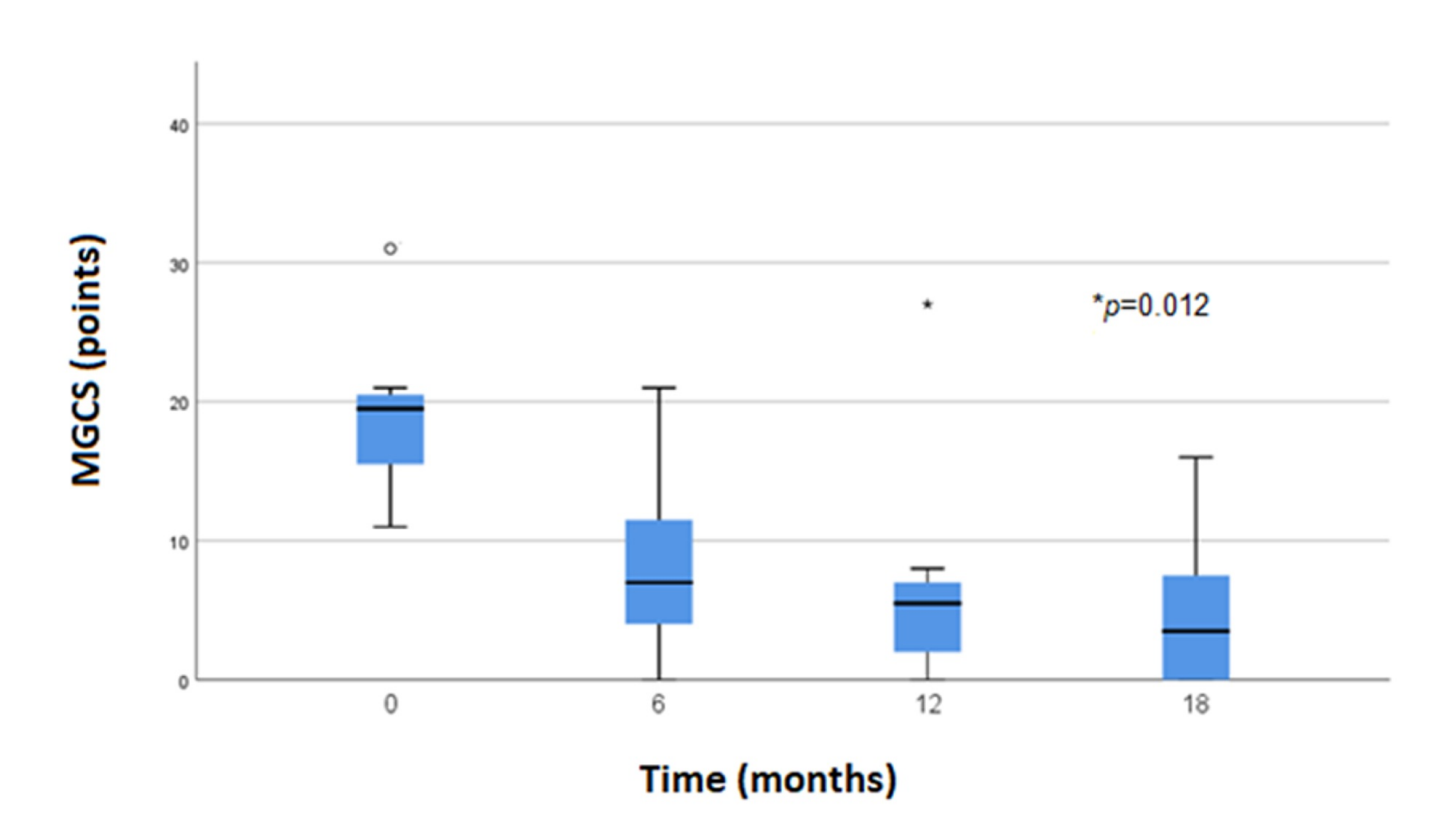
MGCS reduction during follow-up after rituximab therapy. P-value after comparing MGCS median before treatment (0 month) vs MGCS median after 18 months. MGCS - Myasthenia gravis composite score.

**Figure 2:**
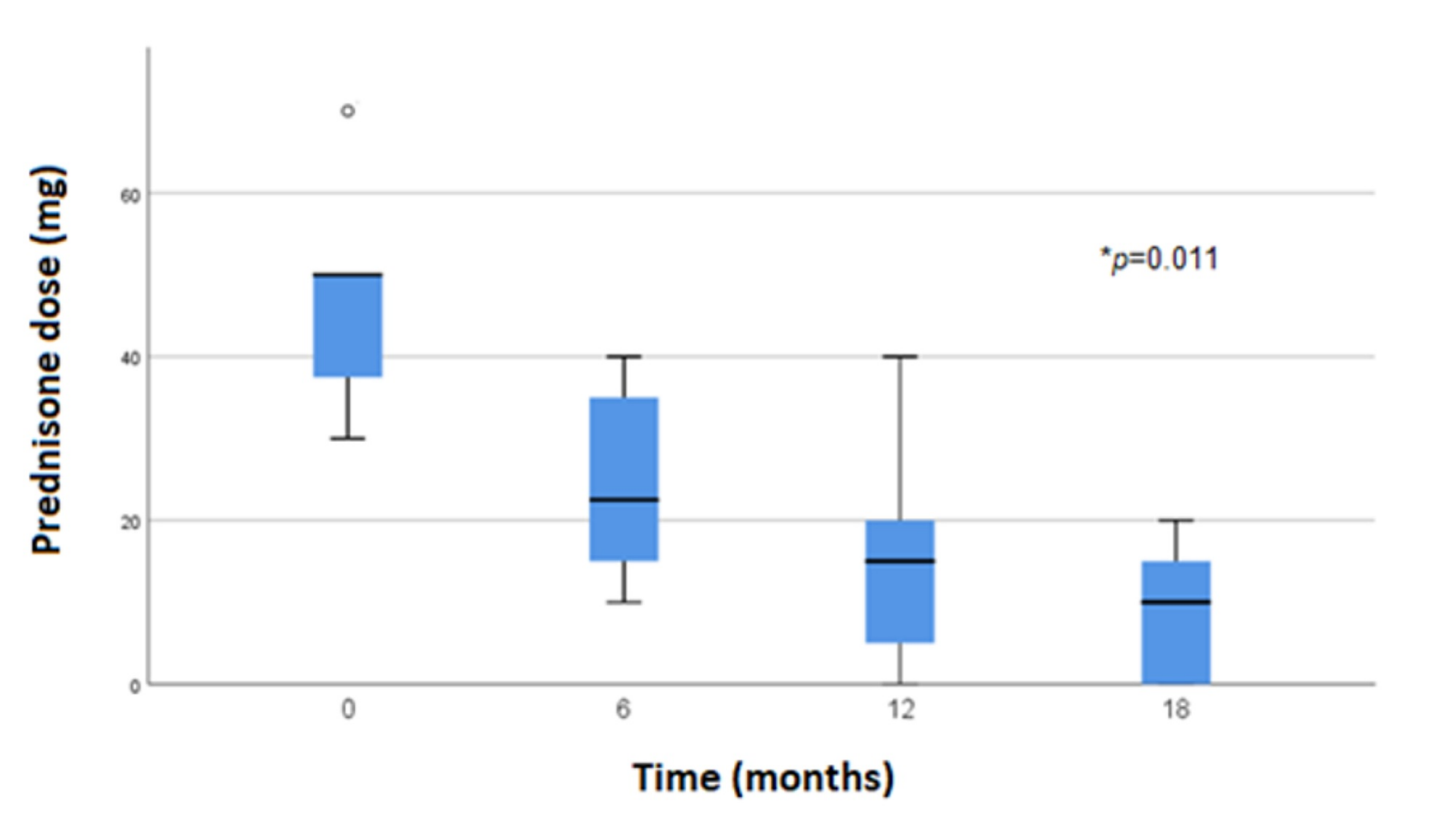
Prednisone dose (mg) reduction during follow-up after rituximab therapy. P-value comparing median prednisone dose before treatment vs median prednisone dose after 18 months.

The median prednisone dose before rituximab treatment was 50 mg (IQR 30-50 mg) and the MGCS median was 19.5 (IQR 11-31). After six and 12-month follow-up, the median prednisone dose was 22.5 and 10 mg, respectively. After 18-month follow-up, only one patient presented with a myasthenic crisis, while the median MGCS was 6 (IQR 0-16) and the median prednisone dose was 10 mg (IQR 0-20 mg). We compared median MGCS and prednisone dose before rituximab treatment vs 18 months after treatment with significative results (MGCS, p=0.012 and prednisone dose, p=0.011) (Figures 1, 2).

All patients (100%) fell into the minimal manifestations (MM-3) MGFA postintervention category in the 18-month evaluation, meaning they had minimal symptoms and yet received cholinesterase inhibitors or other symptomatic therapy and some form of immunosuppression during the last year. There were no adverse effects reported with rituximab therapy during the infusion or follow-up evaluations.

## Discussion

MG has an estimated annual incidence of 8-10 cases per million person-years [1]. Clinical phenotypes correlate with antibody serostatus. Generalized MG is the most common clinical phenotype. Eighty percent of patients have positive AchR antibodies and have a good clinical response to conventional therapy with steroids and immunosuppressants (azathioprine, cyclosporine, mycophenolate, and cyclophosphamide). Severe generalized presentations with early bulbar involvement are mostly related to MusK antibodies, which are present in 1%-14% of MG patients and have a good clinical response to rituximab therapy [2].

Ten to fifteen percent of patients are refractory to conventional treatment [8]. In our MG cohort, only 7% met the definition of refractory MG, which is less than reported in other centers. The most frequent risk factors for refractory MG patients are young age, female gender, thymoma evidence, and MusK antibodies [8]. We did not have any refractory MG patient associated with Musk antibodies.

Rituximab acts as an antibody that binds to CD20 antigen. It has proven to be effective in autoimmune diseases such as rheumatoid arthritis, SLE and Sjogren syndrome. In the last decade, rituximab has proven to be a promising therapy in autoimmune neurological diseases such as N-methyl-d-aspartate (NMDA) encephalitis, neuromyelitis optica, multiple sclerosis, multifocal motor neuropathy and MG. The evaluation of rituximab response in MG is empirical because measurement of AchR antibodies and CD20+ B cells are unhelpful [9,10].

There is little information on the use of rituximab in MG patients. Nonetheless, it has shown to associated with patient improvement in retrospective and prospective case series. Therapy response evaluations after rituximab therapy are different between literature reports. However, all reports evaluate as primary outcome the steroid dose reduction, number of relapses and rescue therapy requirement during follow-up. The most common MG evaluation scores are manual muscle testing (MMT), quantitative MG (QMG) score, MGFA-PIS, and MGCS. Due to the nature of our study, we used the MGCS, which evaluates only clinical parameters [11-13].

Patients with newly diagnosed MG respond faster to initial rituximab therapy and have better scores on follow-up compared to patients with long-lasting MG or those treatment refractory. All our patients had been diagnosed with MG for several years, in addition to being treatment refractory [13,14]. Most papers evaluating refractory MG with rituximab therapy include anti-AchR and anti-MusK patients and show favorable response in both groups, demonstrated by significant steroid dose reduction and lower QMG, MGC, or MMT scores [15]. Most studies do not mention any severe adverse effect related to rituximab therapy. Anti-MusK patients have a more sustained response compared with anti-AchR patients [16]. In our series, all refractory MG patients were positive for AchR antibodies. Moreover, all of them had a statistically significant prednisone dose reduction, as well as in the follow-up MGCSs at 6, 12, and 18 months. Rituximab appears to be well tolerated in MG patients, as none of our patients presented adverse effects during or after the infusion. Pyridostigmine therapy remained similar compared to before rituximab. Some studies report a 15% rate of adverse effects with rituximab, similar to other immunosuppressing therapies [6].

The dose of rituximab used in MG varies between centers. The most common dose schedule is 375 mg/m^2^ once a week for four weeks, followed by 375 mg/m^2^ every six months. An alternative is an induction dose of 1 gram on day 0 and 14, followed by a maintenance dose of 1 gram every six months [10]. Reports mention low-dose rituximab therapy (600 mg every six months for three doses) is enough to control MG symptoms and reduce steroid dose [17,18]. Our patients received rituximab with a 1-gram induction dose on day 0 and 14, followed by a 1-gram maintenance dose every six months.

There is currently no meta-analysis evaluating the efficacy of rituximab in MG due to limited information from clinical trials. The B-cell targeted treatment in MG (BeatMG) trial was a clinical trial that appeared to be negative, although never published [18]. The reason for this was that patients responded to treatment with placebo; however, they had more relapses and myasthenic crisis, so a definite conclusion is still on the air. Most of the reports are case series, although most highlight a good and sustained response with rituximab in MG [19,20].

Limitations of the present study include the retrospective observational design and the small number of patients. Other limitation was the data collection was performed during routine clinical practice. Comparison with other studies is difficult, as rituximab doses and intervals are different, as well as outcome measures. Moreover, most studies are not comparative. Most information comes from case series, which are often biased toward overreporting positive results. Apart from BeatMG, no relevant randomized controlled trials were identified.

## Conclusions

Rituximab appears to be associated with clinical improvement and prednisone dose reduction in patients diagnosed with anti-AchR MG. Our findings need to be interpreted in light of the limitations mentioned.

There is still uncertainty regarding which MG patients benefit the most with rituximab therapy. This may set the background for further well-designed clinical trials to demonstrate efficacy and safety.
